# Estimating the incidence and risk factors of postpartum hemorrhage from the national ENACT network

**DOI:** 10.1038/s44401-026-00088-x

**Published:** 2026-07-30

**Authors:** Malarkodi J. Samayamuthu, Olga Kravchenko, Wei-Hsuan Lo-Ciganic, Eugene M. Sadhu, Seonkyeong Yang, Vanathi Gopalakrishnan, Shyam Visweswaran

**Affiliations:** 1 Department of Biomedical Informatics, University of Pittsburgh, Pittsburgh, PA USA; 2 Department of Family Medicine, University of Pittsburgh, Pittsburgh, PA USA; 3 Division of General Internal Medicine, School of Medicine, University of Pittsburgh, Pittsburgh, PA USA; 4 Center for Pharmaceutical Policy and Prescribing, University of Pittsburgh, Pittsburgh, PA USA; 5 North Florida / South Georgia Veterans Health System; Geriatric Research Education and Clinical Center, Gainesville, FL USA; 6 The Intelligent Systems Program, University of Pittsburgh, Pittsburgh, PA USA

**Keywords:** Diseases, Health care, Medical research, Risk factors

## Abstract

Aggregated counts from electronic health records (EHRs) provide a rich source of real-world data for investigating critical medical conditions such as postpartum hemorrhage (PPH). We used the Evolve to Next-Gen Accrual to Clinical Trials (ENACT) network, a large, federated network of EHRs, to characterize national trends, risk factors, and comorbidities associated with PPH in a cohort of 705,120 women hospitalized for delivery from 2005 to 2022. During this period, there was a statistically significant increase in the incidence of PPH, consistent with previous studies. Asian women had the highest incidence of PPH (10.33%), followed by Black or African American (8.33%) and American Indian or Alaska Native (8.05%) women. In PPH deliveries, the top-ranked risk factor was severe eclampsia (18.51%), the top-ranked comorbidity was operative vaginal delivery (16.98%), and the commonest cause was uterine atony (83.06%). We demonstrated that a large federated EHR network such as ENACT can be used to generate population-level epidemiological findings, albeit with some limitations.

## Introduction

Health sciences research increasingly relies on real-world data to examine disease patterns, evaluate care delivery, and generate evidence at scale. Electronic health records (EHRs), which record patient contacts, treatments, and clinical activities within healthcare systems, are a valuable source of real-world data and are increasingly used in epidemiological, clinical, and translational studies. In this article, we use postpartum hemorrhage (PPH), a critical obstetric condition with significant impact on maternal morbidity and mortality, as a population-level case study to both characterize national epidemiologic patterns and to assess the validity of a country-wide federated EHR data network in the United States (U.S.), the Evolve to Next-Gen Accrual of Patients to Clinical Trials (ENACT) network, population-level research^1^.

Multi-institutional EHR research generally follows one of the two primary approaches: centralized or federated. In the centralized approach, patient-level data from participating institutions are aggregated into a single repository, which enables detailed analyses but often entails delays in data availability and scalability challenges. In contrast, federated networks retain data locally at participating institutions, harmonize it to common standards, and enable rapid access to large volumes of data with lower security risk, though with some limitations on the types of analyses that can be performed. ENACT is one such federated network; other examples include the National Patient-Centered Clinical Research Network (PCORnet)^2^ and the Consortium for Clinical Characterization of COVID-19 by EHR (4CE)^3,4^.

The ENACT network enables users with no programming knowledge to interactively query network data repositories. Data at each site are mapped to either the Informatics for Integrating Biology at the Bedside (i2b2) or the Observational Medical Outcomes Partnership data models, which are linked via the Shared Health Research Information Network (SHRINE) platform^5^. The network comprises 57 data-contributing institutions (ENACT sites) affiliated with academic medical research centers. Data can be queried across core clinical domains, including demographics, diagnoses, procedures, medications, laboratory test results, and visit characteristics. To maintain data currency, each site in the network updates its repository at least once per month. While the SHRINE platform enables querying all sites on the network, not all may respond to every query due to data repository downtime, network traffic, and other technical issues. Furthermore, the set of responding sites may vary with each query, particularly when a large number of queries are run. Additionally, to preserve patient privacy, the SHRINE platform automatically obfuscates query results. While the ENACT network has the potential to accelerate evidence generation through large-scale EHR analysis, the first step is to evaluate the alignment of evidence generated from the network with findings from prior research using patient-level data.

In this study, we used the ENACT network to conduct a population-level epidemiological analysis of PPH. PPH is one of the top five causes of maternal mortality in both resource-abundant and resource-limited countries, although the absolute risk of death from PPH is substantially lower in developed countries. In the U.S., the incidence of PPH has increased from 2.7% in 2000 to 4.3% in 2019, although maternal mortality from PPH has decreased since the 1980s, with a reduction to 1.7 deaths per 100,000 live births in 2009^6–8^. Despite this decline, in 2019, PPH accounted for 21.2% of pregnancy-related deaths in the U.S^9^.

The American College of Obstetricians and Gynecologists defines PPH as either cumulative blood loss of greater than or equal to 1000 mL or blood loss accompanied by signs or symptoms of hypovolemia within 24 hours of childbirth^10^. The criteria apply to both vaginal and cesarean deliveries. PPH is divided into subtypes based on symptom onset. Primary PPH is hemorrhage that occurs between the third stage of labor (i.e., delivery of the placenta) and 24 hours after fetal delivery, and secondary PPH is bleeding that occurs more than 24 hours after delivery and up to 12 weeks postpartum. The causes of PPH are summarized by the “Four Ts,” which include tone (e.g., uterine atony), trauma (e.g., laceration, rupture), tissue (e.g., retained products of conception), and thrombin (e.g., coagulopathies). Uterine atony has been identified as the most common cause, accounting for approximately 80% of PPH deliveries^11,12^. Treatment of PPH requires prompt recognition and management of the cause of hemorrhage, involving a well-coordinated team effort to address it. Surgical interventions, such as hysterectomy, are used only as a last resort. Key maternal risk factors for PPH include intrinsic factors such as placenta previa, polyhydramnios, multiple gestation, and uterine leiomyomas, or factors related to circumstances around labor and delivery, such as severe preeclampsia, chorioamnionitis or endometritis, prolonged labor, and prior cesarean^6,13^. Additionally, comorbidities and other clinical factors such as pregestational and gestational diabetes, asthma, chronic hypertension, antepartum hemorrhage, or abruption may further increase the risk of PPH. Prevention of PPH includes identifying and managing risk factors and associated comorbidities, as well as active management of labor. Identifying the risk factors is crucial for targeted preventative measures and improving maternal health outcomes^14^.

We used the SHRINE platform to obtain a comprehensive set of counts of hospitalized deliveries and PPH, which enabled estimation of the proportion of deliveries affected by PPH, associated risk factors, and comorbidities. A repeated annual cross-sectional analysis was conducted to examine trends in PPH incidence and the burden of risk factors and comorbidities among affected women. We hypothesized that insights from the ENACT network would be consistent with findings from prior research using patient-level data, such as data collected by the Agency for Healthcare Research and Quality’s (AHRQ’s) in the National (Nationwide) Inpatient Sample (NIS)^6^. Our study is vital for validating the ENACT network’s effectiveness in generating real-world health sciences data, particularly for critical conditions such as PPH.

Overall, this study has two complementary objectives: (1) to characterize national trends, risk factors, and comorbidities associated with postpartum hemorrhage using aggregated EHR data, and (2) to examine how a large federated EHR network such as ENACT can be used to generate population-level epidemiological insights. We aimed to demonstrate both the epidemiological findings that can be obtained and the practical strengths and constraints of ENACT for population-level research.

## Results

Data for this study were obtained from the ENACT network and consist of counts of women hospitalized for delivery, experienced PPH, and had various risk factors and comorbidities. The raw dataset comprised 705,120 women hospitalized for delivery, collected from eight ENACT active sites between 2005 and 2022. Though there are more sites in the network, we limited our analysis to data from those that provided results for all queries.

The eight sites included in the analyses collectively represented all four U.S. Census regions, with two contributing sites per region. Specifically, the regions were Northeast, Midwest, West, and South, each represented by two sites.

### Study cohort characteristics

Table 1 shows the age and racial characteristics, risk factors for PPH, and maternal comorbidities of all women hospitalized for delivery, with PPH, and without PPH deliveries. Most deliveries occurred in women aged 18–54 years (85.16%), and nearly all PPH deliveries (98.63%) also occurred within this age range. Due to querying constraints, maternal age refers to the woman’s age at the time of querying the network, not the age at delivery. Because the age at delivery was unavailable, the age-based breakdown provides only approximate estimates of the percentages of PPH within age groups. Hence, our analyses did not use age breakdown data (see the Methods section). White women had the highest deliveries, with 475,215 (67.54%) total deliveries and 37,345 (66.13%) PPH deliveries. Black or African American women had 88,470 (12.55%) total deliveries and 7370 (13.05%) PPH deliveries.

**Table 1:** Demographic characteristics, risk factors, and comorbidities of all women hospitalized for delivery, those with postpartum hemorrhage (PPH), and those without PPH

	All	With PPH	Without PPH	*P* value
All women	705,120	56,475	648,645	
Age*				
<18 years	89,340 (12.67)	0	89,340 (13.77)	<0.001
18–54 years	600,490 (85.16)	55,700 (98.63)	544,790 (83.99)	<0.001
>55 years	15,220 (2.16)	745 (1.32)	144,75 (2.23)	<0.001
Maternal race				
American Indian or Alaska Native	3105 (0.44)	250 (0.44)	2855 (0.44)	<0.001
Asian	40,850 (5.79)	4220 (7.47)	36,630 (5.65)	<0.001
Black or African American	88,470 (12.55)	7370 (13.05)	81,100 (12.50)	<0.001
Multiple races	4380 (0.62)	350 (0.62)	4030 (0.62)	<0.001
Native Hawaiian or other Pacific Islander	1550 (0.22)	115(0.20)	1435 (0.22)	<0.001
Unknown	92,085 (13.06)	6940 (12.29)	85,145 (13.13)	0.021
White	476,215 (67.54)	37,345 (66.13)	438,870 (67.66)	<0.001
Risk factor				
Prior cesarean (no placenta previa or accreta)	113,240 (16.06)	11,315 (20.04)	101,925 (15.71)	<0.001
Placenta previa or accreta	48,160 (6.83)	8450 (14.96)	39,710 (6.12)	<0.001
Severe eclampsia	34,305 (4.87)	6350 (11.24)	27,955 (4.31)	<0.001
Polyhydramnios	16,520 (2.34)	2360 (4.18)	14,160 (2.18)	<0.001
Chorioamnionitis or endometritis	64,295 (9.12)	11,405 (20.19)	52,890 (8.15)	<0.001
Multiple gestation	31,375 (4.45)	4680 (8.29)	26,695 (4.12)	<0.001
Uterine leiomyomas	33,315 (4.72)	3930 (6.96)	29,385 (4.53)	<0.001
Comorbidity				
Obesity	138,265 (19.61)	13,580 (24.05)	124,685 (19.22)	<0.001
Pregestational diabetes	39,900 (5.66)	4580 (8.11)	35,320 (5.45)	<0.001
Gestational diabetes	82,275 (11.67)	9005 (15.95)	73,270 (11.30)	<0.001
Asthma	76,320 (10.82)	7490 (13.26)	68,830 (10.61)	<0.001
Chronic hypertension	75,215 (10.67)	9000 (15.94)	66,215 (10.21)	<0.001
Antepartum hemorrhage or abruption	60,265 (8.55)	8690 (15.39)	51,575 (7.95)	<0.001
Operative vaginal delivery	20,055 (2.84)	3405 (6.03)	16,650 (2.57)	<0.001
Primary cesarean	182,275 (25.85)	191,85 (33.97)	163,090 (25.14)	<0.001

The percentages (in parentheses) for maternal race, risk factors, and comorbidities are “column” percentages, which denote the percentage of each subgroup within all, with PPH, and without PPH delivery groups.

Table 1 also reports the breakdown of PPH rates among women with various risk factors and comorbidities. Among risk factors, women with prior cesareans (no placenta previa or accreta) had a PPH rate of 20.04%, while those with placenta previa or accreta had a rate of 14.96%. Women with severe eclampsia had a rate of 11.24%, women with chorioamnionitis or endometritis had a rate of 20.19%, women with polyhydramnios had a rate of 4.18%, and women with multiple gestation had a rate of 8.29%. Among comorbidities, the highest rate of PPH occurred in women with primary cesarean (33.97%) and women with obesity (24.05%).

### Incidence, causes, and interventions in postpartum hemorrhage

Table 2 summarizes the incidence of PPH across racial groups, the number of risk factors, and the number of comorbidities. Among 705,120 women hospitalized for delivery between 2005 and 2022, 56,475 were complicated by PPH, yielding an overall crude incidence rate of 8.01%. PPH incidence varied substantially across racial groups. White women, the reference group, had a crude incidence rate of 7.84%, whereas Asian women experienced a significantly higher rate at 10.33% (IRR = 1.32, CI [1.28, 1.36]), followed by Black or African American women (8.33%, IRR = 1.06, CI [1.04, 1.09]).

**Table 2:** Absolute risk (crude incidence) rates and crude incidence rate ratios (IRRs) of women with postpartum hemorrhage (PPH) by maternal race, number of risk factors, and number of comorbidities

	All	With PPH	Absolute risk for each subgroup (crude incidence rate, %)	Crude incidence rate ratio (IRR)
All women	705,120	56,475	8.01	
Maternal race				
American Indian or Alaska Native	3105	250	8.05	1.03 [0.91 – 1.16]
Asian	40,850	4220	10.33	1.32 [1.28 – 1.36]
Black or African American	88,470	7370	8.33	1.06 [1.04 – 1.09]
Multiple races	4380	350	7.99	1.02 [0.92 – 1.13]
Native Hawaiian or other Pacific Islander	1550	115	7.42	0.95 [0.79 – 1.14]
Unknown	92,085	6940	7.54	0.96 [0.94–0.99]
White (reference)	476,215	37,345	7.84	1.00 (Reference)
Number of risk factors				
Women with 0 risk factors	440,125	22,780	5.18	1.00 (Reference)
Women with at least 1 risk factor	265,020	33,755	12.74	2.46 [2.42 – 2.50]
Women with at least 2 risk factors	182,745	24,995	13.68	2.64 [2.60 – 2.69]
Number of comorbidities				
Women with 0 comorbidity	327,725	17,150	5.23	1.00 (Reference)
Women with at least 1 comorbidity	377,425	39,380	10.43	1.99 [1.96–2.03]
Women with at least 2 comorbidities	246,940	27,060	10.96	2.09 [2.05 – 2.13]

The crude incidence rate refers to the percentage of women with PPH among all women hospitalized for delivery within each subgroup. The crude IRR is obtained by dividing a subgroup’s crude incidence rate by a reference subgroup’s rate. The reference subgroups are White women (for race), those with no PPH risk factors (for number of risk factors), and those with no comorbidities (for number of comorbidities).

These racial patterns are consistent with findings from prior research. A recent systematic review and meta-analysis reported PPH odds ratios of 1.33 for Asian women and 1.16 for Black women relative to White women^15^. A statewide study in Hawaii found adjusted odds ratios of 1.45 for Asian women and 1.40 for Native Hawaiian and Other Pacific Islander women compared with White women^16^. In a large Southeastern medical center study, PPH occurred in 7% of vaginal deliveries among White women, 7.2% among Black women, and 7.9% among Native American women^17^. These results align with the elevated incidence observed in our cohort for Asian women and the more modest elevation for Black women.

Women with at least one risk factor had a PPH incidence rate of 12.74% (IRR = 2.46, CI [2.42, 2.50]), which was more than twice the PPH rate of those with no risk factors (reference group, 5.18%), and women with at least two risk factors had the highest PPH incidence rate of 13.68% (IRR = 2.64, CI [2.60, 2.69]) compared to the reference group. Women with at least one comorbidity had a PPH incidence rate of 10.43% (IRR = 1.99, CI [1.96, 2.03]), which was significantly higher than the PPH rate of those with no comorbidities (reference group, 5.23%), and women with at least two comorbidities had the highest PPH incidence rate of 10.96% (IRR = 2.09, CI [2.05, 2.13]) compared to the reference group. These findings demonstrate a clear increase in PPH risk as risk factors and comorbidities accumulate.

**Fig. 1:**
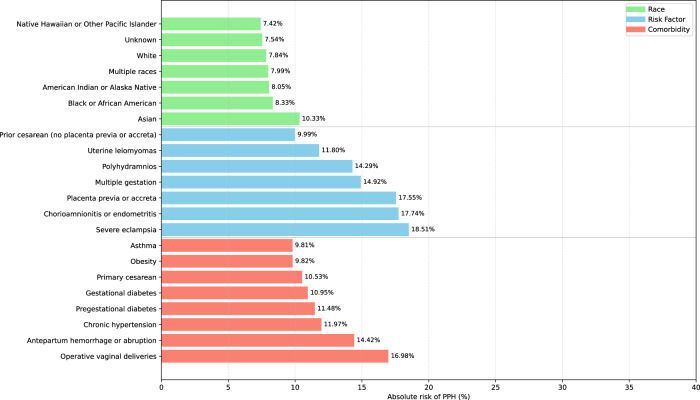
Absolute risk (crude incidence) rates of postpartum hemorrhage (PPH). PPH rates (given as percentages) are ranked in ascending order by race (green), risk factor (blue), and comorbidity (red).

Figure 1 presents the breakdown of PPH rates by race, risk factors, and comorbidities. Among risk factors, women with prior cesarean (no placenta previa or accreta) had the lowest PPH rate of 9.99%, while those with severe eclampsia, chorioamnionitis or endometritis, and placenta previa or accreta had the highest rates of 18.51%, 17.74%, and 17.55%, respectively. Among comorbidities, the lowest rates of PPH were observed in women with asthma (9.81%) and obesity (9.82%), and the highest rates occurred in women with operative vaginal deliveries (16.98%) and with antepartum hemorrhage or abruption (14.42%).

Table 3 presents the causes of PPH and interventions. Among the causes, uterine atony was the most common, accounting for 46,910 PPH deliveries, followed by tissue-related causes such as retained placenta, which accounted for 13,450 PPH deliveries. Trauma was identified as the cause in 8830 PPH deliveries, and thrombin-induced PPH occurred in 1480 PPH deliveries. Among interventions, a surgical procedure was performed in 5940 deliveries, of which 920 were hysterectomies. Manipulative procedures (e.g., manual removal of retained placenta or blood clots) were performed in 7470 PPH deliveries, and blood transfusions were given in 3390 PPH deliveries. The most common intervention was the administration of PPH-specific medications, which occurred in 19,680 deliveries.

**Table 3:** Postpartum hemorrhage (PPH) causes and interventions

Cause	PPH
Atony	46,910 (83.06)
Trauma	8830 (15.64)
Tissue-related	13,450 (23.82)
Thrombin-induced	1480 (2.62)
Intervention	PPH
Hysterectomy	920 (1.63)
Surgical procedures (including hysterectomy)	5940 (10.52)
Manipulative procedures	7470 (13.23)
Blood transfusion	3390 (6.00)
Medications	19,680 (34.85)

The reported percentages for the causes (interventions) do not total 100% because a woman with PPH may have more than one cause and receive more than one intervention.

### Trends in postpartum hemorrhage

**Fig. 2:**
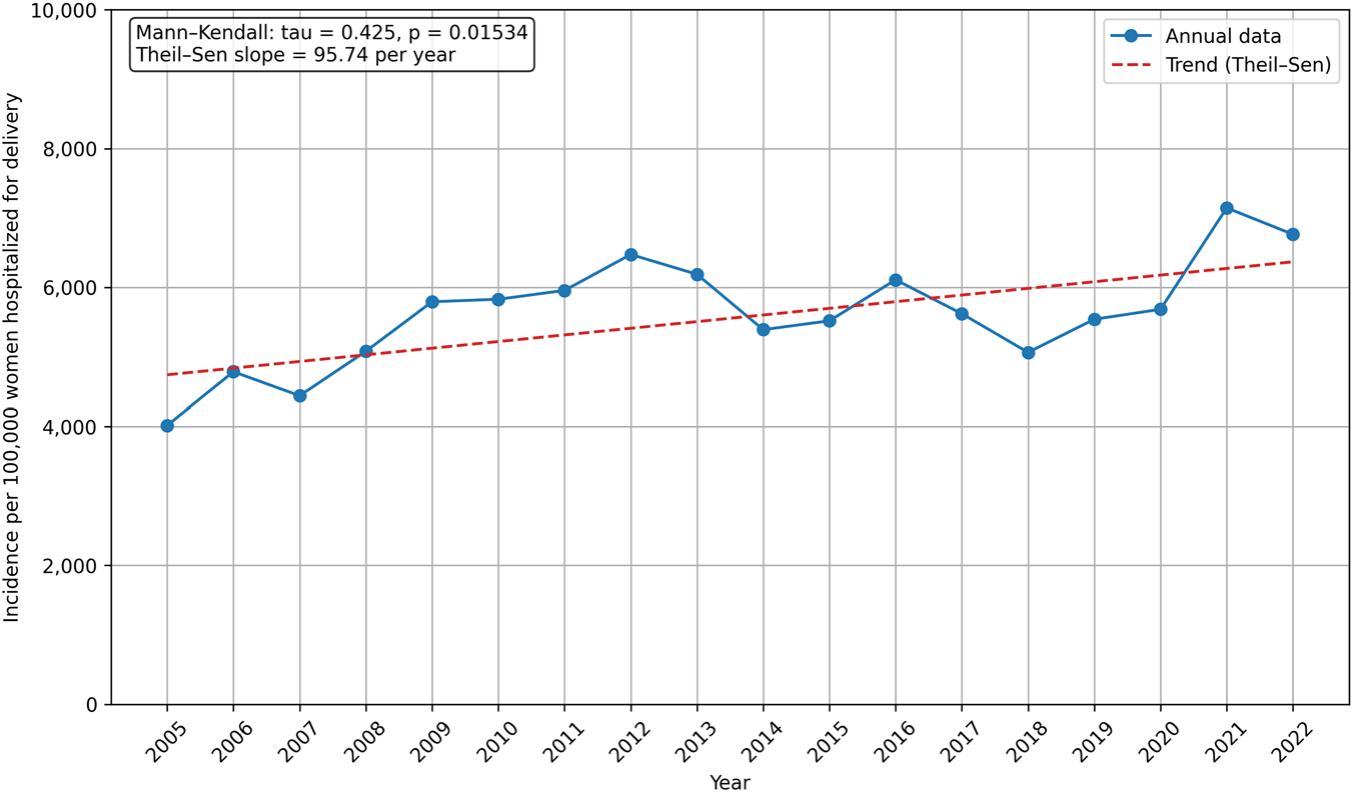
The trend in postpartum hemorrhage (PPH) incidence from 2005 to 2022. The incidence of PPH per 100,000 women hospitalized for delivery (blue) and the corresponding trend line (dashed red).

Figure 2 shows that during the period from 2005 to 2022, there was a statistically significant increase in the incidence of PPH (*P*_trend_ < 0.001). The incidence of PPH per 100,000 women hospitalized for delivery increased from ~4015 to 6764 per 100,000 women (68% increase over the study period). For context, analysis of AHRQ’s NIS data (representing a 20% annual sample of all U.S. hospitalizations) showed an increase in PPH from 2.7% in 2000 to 4.3% in 2019^6^. In contrast, our data demonstrated both a higher baseline incidence and a steeper rise, increasing from 4.0% in 2005 to 5.5% in 2019. Furthermore, the upward trajectory continued beyond 2019, with rates reaching 6.8% in 2022.

Several factors may explain the discrepancies. Differences have been noted between medical claims-based data and clinical data in EHRs. The NIS data comprises diagnosis and procedure codes (ICD-9-CM, ICD-10-CM, and ICD-10-PCS) from medical claims, whereas the data in ENACT are obtained from EHRs. A study reported that, although the distribution of most diagnoses was similar between the NIS data and a large inpatient EHR database (Health Fact; Cerner Corporation), obstetric and gynecologic hospitalizations were significantly underrepresented in the EHR data compared with the NIS^18^. This discrepancy may be attributed to the continued reliance of some obstetric and gynecologic services on paper-based medical records as late as the 2010s^18^.

Further, the ENACT sites are large urban academic hospitals, while the NIS includes a national sample of both academic and community hospitals. Some research has shown that the incidence of PPH tends to be higher in large urban hospitals compared to rural or community hospitals. Studies using the NIS data have reported an increased risk of PPH in urban hospitals compared with rural hospitals^19^, although other studies have reported higher odds of PPH in rural low-volume hospitals than in low-volume urban hospitals^20,21^.

### Comorbidities in postpartum hemorrhage

**Fig. 3:**
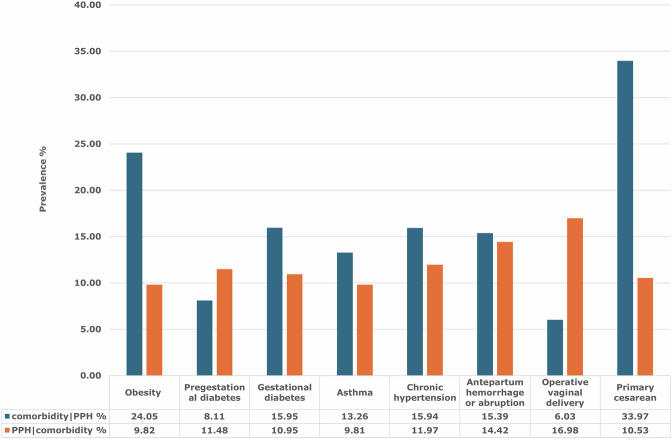
Comorbidity burden in postpartum hemorrhage (PPH). The prevalence of comorbidities in PPH deliveries (blue) and the reciprocal prevalence of PPH in comorbid conditions (orange).

Figure 3 illustrates the prevalence of various comorbidities in PPH deliveries and the reciprocal prevalence of PPH within those comorbid conditions. Comorbidities such as obesity, gestational diabetes, asthma, chronic hypertension, and antepartum hemorrhage or abruption have a high prevalence in PPH deliveries. The reciprocal prevalence of PPH is particularly high among women with antepartum hemorrhage or abruption (14.42%), operative vaginal deliveries (16.98%), and chronic hypertension (11.97%). While primary cesarean has the highest prevalence of PPH among comorbid conditions (33.97%), the prevalence of PPH in women undergoing primary cesarean is relatively lower (10.53%). Conversely, obesity, with a high prevalence in PPH deliveries (24.05%), also shows a significant reciprocal PPH prevalence (9.82%).

### Regional variation in postpartum hemorrhage

Results showing regional variation across the U.S. Census regions are provided in the Supplementary information material. Demographic characteristics, risk factors, and comorbidities of women hospitalized for delivery, women with PPH, and women without PPH across the four regions are provided in Table S2 of the Supplementary information. Absolute risk (crude incidence) rates and crude incidence rate ratios (IRRs) of women with PPH across the four regions are provided in Tables S3-S6 of the Supplementary information. Supplementary Fig. S1 shows a comparative relative risk analysis. Compared with the Northeast, the Midwest had a similar risk (RRR = 1.00, CI = 0.98–1.02), the West had a significantly higher risk (1.07, CI = 1.05, 1.10), and the South had a significantly lower risk (0.73, CI = 0.71, 0.74). These results are in alignment with a study using NIS data that found that, compared with the Northeast, the Midwest and the West had higher adjusted odds of PPH (AOR = 1.20; CI = 1.06–1.35 and [AOR = 1.13; CI = 1.01–1.28, respectively) while the South had lower adjusted odds of PPH (AOR = 0.80; CI = 0.71–0.89)^22^.

## Discussion

One of the objectives of this study was to assess the trends in PPH, risk factors, and comorbidities in the U.S. using aggregated counts from the ENACT network. Our analysis revealed a rising trend in PPH, consistent with the trend observed in a previously published study covering the 2009–2019 period. This study relied on the NIS data, which comprises a 20% sample of all delivery hospitalizations in the U.S. and is collected annually by the AHRQ’s Healthcare Cost and Utilization Project^6^. By including data from before 2009 and after 2019, our study corroborates previously observed trends and shows that the upward trajectory of PPH continues beyond the earlier study period. Given that PPH is life-threatening, our findings underscore the urgent need to identify women at risk of PPH for timely prevention and management.

Another objective of this study was to validate the utility of the data in the ENACT network for conducting research efficiently. Despite the data being available only as aggregate counts, we established reproducible data-quality procedures and analysis workflows that confirm the reliability of the network data for similar analyses. Our querying methods were refined iteratively after discussions with the ENACT technical team to ensure data accuracy. This validation is significant because it demonstrates that the network’s data can be a valuable resource for future research.

Based on the incidence rates of PPH across the various races in our results, we found that the Asian women had the highest rate of PPH (10.33%), followed by Black or African American women (8.33%), and American Indian or Alaska Native women (8.05%). Our findings are consistent with prior studies that confirmed similar disparities using more rigorous statistical methods applied to patient-level EHR data and medical charts^16,23,24^. Our analyses identified the commonest risk factor as severe eclampsia (18.51% in PPH) and the commonest comorbidity as operative vaginal delivery (16.98% in PPH). In concordance with the results obtained from AHRQ’s NIS data^6^, our results show that uterine atony is the commonest cause of PPH.

Overall, our analysis yields actionable findings regarding racial disparities, showing that Asian, Black or African American, American Indian or Alaska Native women have an increasing prevalence of PPH. The rank ordering of comorbidities and risk factors also emphasizes the importance of identifying and managing multiple gestations, prior cesarean, gestational diabetes, and chronic hypertension. These comorbidities and risk factors can help to inform future studies in designing personalized risk prediction models for PPH.

Our study has several limitations. First, to preserve patient privacy, the ENACT network applies obfuscation to the returned counts (i.e., the returned count is within ±10 of the true count). This obfuscation may introduce some degree of inaccuracy in the reported counts in our study, though when the counts are large (in the tens or hundreds of thousands), obfuscation likely did not affect the accuracy of the results. In the future, the network plans to reduce the degree of obfuscation to enable analyses involving rare events or narrowly defined subpopulations.

Second, the technology used in the ENACT network counts the number of unique patients meeting the query criteria, which may lead to undercounting when multiple occurrences of the same diagnostic code are present. For instance, a count from a query may not account for second, third, or subsequent deliveries in the same woman, which may lead to an underestimation of the number of deliveries, especially when the query spans many years. Depending on the research question, this can be mitigated by choosing an appropriate timeframe for querying the condition of interest.

Third, the diagnosis of PPH was based solely on ICD-9-CM and ICD-10-CM codes, consistent with prior research using NIS data. Including indicators of blood loss, such as changes in hematocrit or estimated blood loss based on the magnitude of the hematocrit drop, could improve the accuracy of identifying PPH in EHR data. However, due to limitations of the SHRINE platform, we were unable to obtain actual hematocrit values, hematocrit changes, or vital signs.

Fourth, we used only ICD-9-CM and ICD-10-CM codes for procedures, following methodologies established in previous studies. Typically, all sites map their procedures to Current Procedural Terminology (CPT-4) codes, but not necessarily to ICD codes. The exclusion of CPT-4 codes may result in incomplete data capture, potentially missing certain delivery procedures.

Fifth, this study is subject to limitations related to the operational complexity of conducting analyses within a federated aggregated data network. Each additional stratification or sensitivity analysis requires executing many distinct queries, resulting in a substantial query burden. As a result, the number of sensitivity analyses that can be practically performed in a single study may be limited. This constraint was an important methodological consideration in the design of our analyses.

Finally, due to the lack of patient-level data, we could not study women longitudinally, infer temporal relationships, or identify confounding factors to infer causality. Using aggregated count data to evaluate correlations among PPH, risk factors, and comorbidities does not establish causal relationships. Additionally, drawing race-based conclusions is challenging since the counts of minority groups (e.g., American Indian or Alaska Native, Native Hawaiian or Other Pacific Islander) are small.

This case study illustrates the use of aggregated counts from EHRs from a federated network of sites across the U.S. to assess trends in PPH across racial and age groups. We observed a clear increase in PPH incidence from 2005 to 2022, consistent with prior research. Furthermore, aggregated counts were sufficient to assess maternal risk factors and comorbidity incidence rates, and the findings were concordant with prior research. Data in the ENACT network are updated regularly, enabling rapid access to data for assessing recent trends in critical conditions such as PPH. Our findings underscore the need for better approaches to more accurately identify women at an elevated risk for PPH while accounting for diverse racial groups. In the future, the limitations of aggregated data will be overcome by conducting studies on cohorts of patients with patient-level data (e.g., the global maternal and newborn health eCohorts^25^) that will yield more precise data to assess trends in PPH and associated risk factors and comorbidities.

## Methods

### Data sources

We used the SHRINE platform to query the ENACT network to obtain patient counts. The EHR data in the network is de-identified to ensure patient privacy and comply with Health Insurance Portability and Accountability (HIPAA) regulations, and SHRINE returns aggregate counts of patients meeting query criteria. To ensure privacy, SHRINE automatically obfuscates the patient count returned by a query by adding or subtracting up to 10 and then rounding the result to the nearest multiple of five^26^. Due to obfuscation, the same query may return slightly different counts when rerun, though these variations are typically negligible, especially when the counts are high. To comply with ENACT data use agreements and institutional privacy requirements, individual contributing sites are not identified in the analyses. The University of Pittsburgh’s Institutional Review Board (IRB) approved the use of the ENACT network for a wide range of research under protocol number STUDY19080059.

### Study design and data extraction

First, we identified women hospitalized for delivery during this time period using the International Classification of Diseases, Ninth and Tenth Revisions, Clinical Modification (ICD-9-CM and ICD-10-CM) diagnosis and procedure codes (see Table S1 of the Supplementary information). Next, within this cohort, we identified women with PPH using ICD-9-CM and ICD-10-CM codes. Women who did not have PPH served as a comparator group. We opted to rely solely on ICD codes for the diagnosis of PPH for several reasons, including prior research that used NIS data that relied on ICD codes, limitations of the SHRINE platform (which did not allow for calculating estimated blood loss by assessing the magnitude of the drop in hematocrit values), and ICD codes have been reported to have high specificity for PPH^27,28^. For these three cohorts (all women hospitalized for delivery, women with PPH, and women without PPH), we ran queries to quantify risk factors and comorbidities, and we ran additional queries to quantify causes and interventions in the PPH cohort.

We also conducted repeated annual cross-sectional analyses using counts aggregated across all selected sites from 2005 to 2022. For this, we ran queries to identify women hospitalized for delivery and women with PPH each calendar year.

The queries were submitted to all sites on the ENACT network. To be included in the final analysis, sites were required to return complete results for all submitted queries. Eight ENACT sites met these requirements.

### Outcomes

Our primary outcome of interest was the annual incidence rate of PPH among delivery hospitalizations, focusing on examining trends in incidence rates over the entire study period. Because of the potential limitations of obfuscated data, we compared demographics, risk factors, and comorbidities between the two groups: deliveries with PPH and deliveries without PPH. The demographics included women 15–54 years old stratified by race (American Indian or Alaska Native, Asian, Black or African American, multiple races, Native Hawaiian or Other Pacific Islander, unknown, and White). Due to constraints in constructing queries in SHRINE, obtaining an individual’s age at a specific moment in the past was not possible. Thus, the maternal age we obtained was the woman’s age at the time of querying the network, not the age at delivery. Therefore, maternal age was not considered as a variable in our analyses.

We assessed several maternal risk factors, including prior cesarean (no placenta previa or accreta), placenta previa or accreta, severe preeclampsia, polyhydramnios, chorioamnionitis or endometritis, multiple gestation, and uterine leiomyomas. In addition, we assessed several comorbidities and other clinical conditions, including obesity, pregestational diabetes, gestational diabetes, asthma, chronic hypertension, antepartum hemorrhage or abruption, operative vaginal delivery, and primary cesarean (or first cesarean delivery). We categorized the causes of PPH according to the “Four Ts” (i.e., tone, trauma, tissue, thrombin) as secondary outcomes. Furthermore, we assessed surgical and medical PPH interventions, such as surgical procedures, including hysterectomy, manipulative procedures, blood transfusion, and medications (see Table S1 of the Supplementary information). The PPH incidence, risk factors, and comorbidities were reported as counts and proportions.

### Data analyses

The trends in PPH incidence rates were examined within subgroups defined by race, presence of maternal risk factors, and presence of comorbidities. To assess the significance of the trend in PPH incidence over time, we employed the non-parametric Mann–Kendall trend test^29^. The Mann–Kendall test was chosen for its robustness against data irregularities and its ability to analyze trends without assuming a specific data distribution. Further, we assessed the incidence of PPH in each risk factor and comorbidity subgroup.

We computed the prevalence of comorbidities for women who had PPH deliveries (comorbidity | PPH), as well as the reciprocal prevalence of PPH delivery given these comorbidities (PPH | comorbidity). Data analyses were conducted using Microsoft Excel version 4.3.2 and Python version 3.7.3.

## Supplementary information

Supplementary Information

## Data Availability

The datasets generated and analyzed during the current study are not publicly available due to patient privacy regulations, but are available from the corresponding authors on reasonable request.

## Code availability

The underlying code for this study is not publicly available but may be made available to qualified researchers on reasonable request from the corresponding authors.
