# Supplementary material for: Estimating the incidence and risk factors of postpartum hemorrhage from the national ENACT network
Source: Npj Health Syst. 2026 Jul 30;3:65. doi: 10.1038/s44401-026-00088-x (PMC13424135; doi:10.1038/s44401-026-00088-x)

**Supplementary information**

Samayamuthu MJ, Kravchenko O, Lo-Ciganic W-H, Sadhu EM, Yang S, Gopalakrishnan V, Visweswaran S. Estimating the incidence and risk factors of postpartum hemorrhage from the national ENACT network.

**Table S1. Diagnosis codes, procedure codes, and medication codes used in the analyses**

| **Primary outcomes** | **ICD-9-CM diagnosis codes** | **ICD-9-CM procedure codes** | **ICD-10-CM diagnosis codes** | **ICD-10-PCS procedure codes** |
| --- | --- | --- | --- | --- |
| Delivery hospitalization | 650  669.5*  669.7*  V27* | 72*  73.22  73.59  73.6  74.0  74.1  74.2  74.3  74.4  74.99 | O80  O82  Z37* | 10E0XZZ  10E0XZ0  10D00Z1  10D00Z2  10D07Z3  10D07Z4  10D07Z5  10D07Z6  10D07Z7  10D07Z8 |
| Postpartum hemorrhage (PPH) | 666* |  | O72* |  |

*All child (downstream) codes are included.

| **Risk factors** | **ICD-9-CM diagnosis codes** | **ICD-9-CM procedure codes** | **ICD-10-CM diagnosis codes** | **ICD-10-PCS procedure codes** |
| --- | --- | --- | --- | --- |
| Prior cesarean | 654.2* |  | O34.21* |  |
| Placenta previa or accreta | 641.0*  641.1*  667* |  | O43.2*  O44* |  |
| Severe preeclampsia | 642.5* |  | O14.1*  O14.20  O14.24  O14.25 |  |
| Polyhydramnios | 657.0*  761.3 |  | O40* |  |
| Chorioamnionitis or endometritis | 615.9  658.4*  659.2*  670* |  | N71.9  O41.1*  O75.2  O86.12 |  |
| Multiple gestation | 651  652.6*  662.3  660.5  V27.2  V27.3  V27.4  V27.5  V27.6  V27.7 |  | O30*  O31  O32.9  O63.2  O66.1  O66.6  Z37.2  Z37.3  Z37.4  Z37.5  Z37.6  Z37.7 |  |
| Uterine leiomyomas | 218* |  | D25* |  |

*All child (downstream) codes are included.

| **Comorbidities** | **ICD-9-CM diagnosis codes** | **ICD-9-CM procedure codes** | **ICD-10-CM diagnosis codes** | **ICD-10-PCS procedure codes** |
| --- | --- | --- | --- | --- |
| Obesity | 278.0*  649.1*  793.91  V85.3*  V85.4* |  | E66.0*  E66.1  E66.2  E66.8  E66.9  O91.22  Z68.3  Z68.4 |  |
| Pregestational diabetes | 249*  250*  648.0* |  | E08*  E09*  E10*  E11*  E13*  O24.0*  O24.1*  O24.3*  O24.8*  O24.9* |  |
| Gestational diabetes | 648.8* |  | O24.4* |  |
| Asthma | 493* |  | J45* |  |
| Chronic hypertension | 401*  402*  403*  404*  405*  642.0*  642.1*  642.2*  642.7* |  | I10  I11  I12*  I13*  I15*  I16*  N26.2  O10*  O11* |  |
| Antepartum hemorrhage or abruption | 641.2*  641.3*  641.8*  641.9* |  | O45.0*  O45.8*  O45.9*  O46.0*  O46.8*  O46.9*  O67* |  |
| Operative vaginal delivery | 669.5* |  |  | 10D07Z3  10D07Z4  10D07Z5  10D07Z6  10D07Z7  10D07Z8 |
| Primary cesarean | 669.7* | 74.0  74.1  74.2  74.4  74.99 | O82 | 10D00Z0  10D00Z1  10D00Z2 |

*All child (downstream) codes are included.

| **Causes** | **ICD-9-CM diagnosis codes** | **ICD-9-CM procedure codes** | **ICD-10-CM diagnosis codes** | **ICD-10-PCS procedure codes** |
| --- | --- | --- | --- | --- |
| Atony | 666.1* |  | O72.1 |  |
| Trauma | 664*  665* |  | 43.2*  O70*  O71* |  |
| Tissue-related | 666.0*  666.2* |  | O72.0  O72.2 |  |
| Thrombin-induced | 666.3 |  | O72.3 |  |

*All child (downstream) codes are included.

| **Interventions** | **ICD-9-CM diagnosis codes** | **ICD-9-CM procedure codes** | **ICD-10-CM diagnosis codes** | **ICD-10-PCS procedure codes** |
| --- | --- | --- | --- | --- |
| Hysterectomy |  | 68.3*  68.4*  68.5*  68.6*  68.7*  68.8  68.9 |  | 0UT90ZZ  0UT94ZZ  0UT97ZZ  0UT98ZZ  0UT9FZZ |
| Surgical procedures (including hysterectomy) |  | 68.3*  68.4*  68.5*  68.6*  68.7*  68.8*  68.9  68.24  68.25  75.93 |  | 04LE*  04LF*  0US90ZZ  0US94ZZ  0US97ZZ  0US98ZZ  0UT90ZZ  0UT94ZZ  0UT97ZZ  0UT98ZZ  0UT9FZZ |
| Manipulative procedures |  | 75.4  75.5*  75.8  75.91  75.94 |  | 0JCB0ZZ  0JCB3ZZ  0UQC*  0UQG*  0UQM*  0US9XZZ  0W3R0ZZ  0W3R3ZZ  0W3R4ZZ  0W3R7ZZ  0W3R8ZZ  10D18Z9  2Y44X5Z |
| Blood transfusion |  | 99.0* (except 99.01)  99.00  99.02  99.03  99.04  99.05  99.06  99.07  99.08  99.09 |  | 30233H1  30233K1  30233L1  30233M1  30233N1  30233P1  30233R1  20233T1  30240H1  30240K1  30240L1  30240M1  30240N1  30240P1  30240R1  30240T1  20243H1  30243K1  30243L1  30243M1  30243N1  30243P1  30243R1  30243T1  30233N0  30233P0  30240N0  30240P0  30243N0  30243P0 |
|  | **RxNorm codes** |  |  |  |
| Medications | 7824  93672  242125  371284  371879  372050  374179  377087  378095  577490  727720  1729374  1789851  1791720 |  |  |  |

*All child (downstream) codes are included.

**Table S2.** Demographic characteristics, risk factors, and comorbidities of all women hospitalized for deliveries, women with postpartum hemorrhage (PPH), and women without PPH for the four U.S. Census regions. The percentages (in parentheses) are "column" percentages, which denote the percentage of each subgroup within all, with PPH, and without PPH delivery groups. Age refers to the age of women at the time of the query rather than the age at delivery.

|  | **All** | **With PPH** | **Without PPH** |
| --- | --- | --- | --- |
| **Northeast** |  |  |  |
| Total | **206,485** | **18,400** | **188,085** |
| Age < 18 years | 1,515 (0.73) | 0 | 1,515 (0.81) |
| Age ≥18 years | 204,840 (99.20) | 18,400 (100.00) | 186,440 (99.13) |
| **Midwest** |  |  |  |
| Total | **130,570** | **11,645** | **118,925** |
| Age < 18 years | 6,805 (5.21) | 0 | 6,805 (5.72) |
| Age ≥18 years | 123,790 (94.81) | 11,645 (100.00) | 112,145 (94.30) |
| **West** |  |  |  |
| Total | **85,090** | **8,145** | **76,945** |
| Age < 18 years | 3,685 (4.33) | 0 | 3,685 (4.79) |
| Age ≥18 years | 81,415 (95.68) | 8,145 (100.00) | 73,270 (95.22) |
| **South** |  |  |  |
| Total | **282,975** | **18,285** | **264,690** |
| Age < 18 years | 77,335 (27.33) | 0 | 77,335 (29.22) |
| Age ≥18 years | 205,635 (72.67) | 18,285 (100.00) | 187,350 (70.78) |

**Table S3.** Absolute risk (crude incidence) rates and crude incidence rate ratios (IRRs) of women with postpartum hemorrhage (PPH) by maternal race, number of risk factors, and number of comorbidities for the Northeast region.

| **Northeast** | **All** | **With PPH** | **Absolute risk for each subgroup (crude incidence rate, %)** | **Crude incidence rate ratio (IRR)** |
| --- | --- | --- | --- | --- |
| All women | 206,485 | 18,400 | 8.91 |  |
| **Maternal Race** |  |  |  |  |
| American Indian or Alaska Native | 900 | 85 | 9.44 | 1.15 |
| Asian | 14,600 | 1,615 | 11.06 | 1.35 |
| Black or African American | 28,800 | 2,930 | 10.17 | 1.24 |
| Multiple Race | 0 | 0 | 0 | 0 |
| Native Hawaiian or Other Pacific Islander | 280 | 0 | 0 | 0 |
| Unknown (No Information) | 21,965 | 2,295 | 10.45 | 1.27 |
| White | 139,795 | 11,490 | 8.22 | 1.00 (Reference) |
| **Risk Factor** |  |  |  |  |
| Women with 0 risk factors | 119,425 | 8,070 | 6.76 | 1.00 (Reference) |
| Women with at least 1 risk factor | 87,060 | 10,350 | 11.89 | 1.76 |
| Women with at least 2 risk factors | 47,635 | 6,260 | 13.14 | 1.94 |
| **Comorbidity** |  |  |  |  |
| Women with 0 comorbidity | 74,250 | 5,385 | 7.25 | 1.00 (Reference) |
| Women with at least 1 comorbidity | 132,230 | 13,025 | 9.85 | 1.36 |
| Women with at least 2 comorbidities | 76,810 | 8,210 | 10.69 | 1.47 |

**Table S4.** Absolute risk (crude incidence) rates and crude incidence rate ratios (IRRs) of women with postpartum hemorrhage (PPH) by maternal race, number of risk factors, and number of comorbidities for the Midwest region.

| **Midwest** | **All** | **With PPH** | **Absolute risk for each subgroup (crude incidence rate, %)** | **Crude incidence rate ratio (IRR)** |
| --- | --- | --- | --- | --- |
| All women | 130,570 | 11,645 | 8.92 |  |
| **Maternal Race** |  |  |  |  |
| American Indian or Alaska Native | 575 | 35 | 6.09 | 0.68 |
| Asian | 5,955 | 645 | 10.83 | 1.22 |
| Black or African American | 14,365 | 1,255 | 8.74 | 0.98 |
| Multiple Race | 265 | 15 | 5.66 | 0.64 |
| Native Hawaiian or Other Pacific Islander | 275 | 15 | 5.45 | 0.61 |
| Unknown (No Information) | 7,645 | 665 | 8.70 | 0.98 |
| White | 100,230 | 8,920 | 8.90 | 1.00 (Reference) |
| **Risk Factor** |  |  |  |  |
| Women with 0 risk factors | 82,120 | 5,690 | 6.93 | 1.00 (Reference) |
| Women with at least 1 risk factor | 48,465 | 5,965 | 12.31 | 1.78 |
| Women with at least 2 risk factors | 40,710 | 5,190 | 12.75 | 1.84 |
| **Comorbidity** |  |  |  |  |
| Women with 0 comorbidity | 48,500 | 3,085 | 6.36 | 1.00 (Reference) |
| Women with at least 1 comorbidity | 82,105 | 8,575 | 10.44 | 1.64 |
| Women with at least 2 comorbidities | 60,860 | 6,650 | 10.93 | 1.72 |

**Table S5.** Absolute risk (crude incidence) rates and crude incidence rate ratios (IRRs) of women with postpartum hemorrhage (PPH) by maternal race, number of risk factors, and number of comorbidities for the West region.

| **West** | **All** | **With PPH** | **Absolute risk for each subgroup (crude incidence rate, %)** | **Crude incidence rate ratio (IRR)** |
| --- | --- | --- | --- | --- |
| All women | 85,090 | 8,145 | 9.57 |  |
| **Maternal Race** |  |  |  |  |
| American Indian or Alaska Native | 855 | 110 | 12.87 | 1.40 |
| Asian | 12,020 | 1,385 | 11.52 | 1.25 |
| Black or African American | 6,965 | 785 | 11.27 | 1.22 |
| Multiple Race | 1,770 | 155 | 8.76 | 0.95 |
| Native Hawaiian or Other Pacific Islander | 550 | 75 | 13.64 | 1.48 |
| Unknown (No Information) | 21,785 | 1,845 | 8.47 | 0.92 |
| White | 41,140 | 3,790 | 9.21 | 1.00 (Reference) |
| **Risk Factor** |  |  |  |  |
| Women with 0 risk factors | 51,650 | 3,560 | 6.89 | 1.00 (Reference) |
| Women with at least 1 risk factor | 33,445 | 4,610 | 13.78 | 2.00 |
| Women with at least 2 risk factors | 21,155 | 3,220 | 15.22 | 2.21 |
| **Comorbidity** |  |  |  |  |
| Women with 0 comorbidity | 37,560 | 2,545 | 6.78 | 1.00 (Reference) |
| Women with at least 1 comorbidity | 47,540 | 5,620 | 11.82 | 1.74 |
| Women with at least 2 comorbidities | 25,275 | 3,310 | 13.10 | 1.93 |

**Table S6.** Absolute risk (crude incidence) rates and crude incidence rate ratios (IRRs) of women with postpartum hemorrhage (PPH) by maternal race, number of risk factors, and number of comorbidities for the South region.

| **South** | **All** | **With PPH** | **Absolute risk for each subgroup (crude incidence rate, %)** | **Crude incidence rate ratio (IRR)** |
| --- | --- | --- | --- | --- |
| All women | 282,975 | 18,285 | **6.46** |  |
| **Maternal Race** |  |  |  |  |
| American Indian or Alaska Native | 775 | 20 | 2.58 | 0.38 |
| Asian | 8,275 | 575 | 6.95 | 1.03 |
| Black or African American | 38,340 | 2,400 | 6.26 | 0.93 |
| Multiple Race | 2,345 | 180 | 7.68 | 1.14 |
| Native Hawaiian or Other Pacific Islander | 445 | 25 | 5.62 | 0.83 |
| Unknown (No Information) | 40,690 | 2,135 | 5.25 | 0.78 |
| White | 195,050 | 13,145 | 6.74 | 1.00 (Reference) |
| **Risk Factor** |  |  |  |  |
| Women with 0 risk factors | 186,930 | 5,460 | 2.92 | 1.00 (Reference) |
| Women with at least 1 risk factor | 96,050 | 12,830 | 13.36 | 4.57 |
| Women with at least 2 risk factors | 73,245 | 10,325 | 14.10 | 4.83 |
| **Comorbidity** |  |  |  |  |
| Women with 0 comorbidity | 167,415 | 6,135 | 3.66 | 1.00 (Reference) |
| Women with at least 1 comorbidity | 115,555 | 12,160 | 10.52 | 2.87 |
| Women with at least 2 comorbidities | 83,995 | 8,890 | 10.58 | 2.89 |

**Figure S1.** Comparison of women with postpartum hemorrhage (PPH) across the U.S. Census regions.


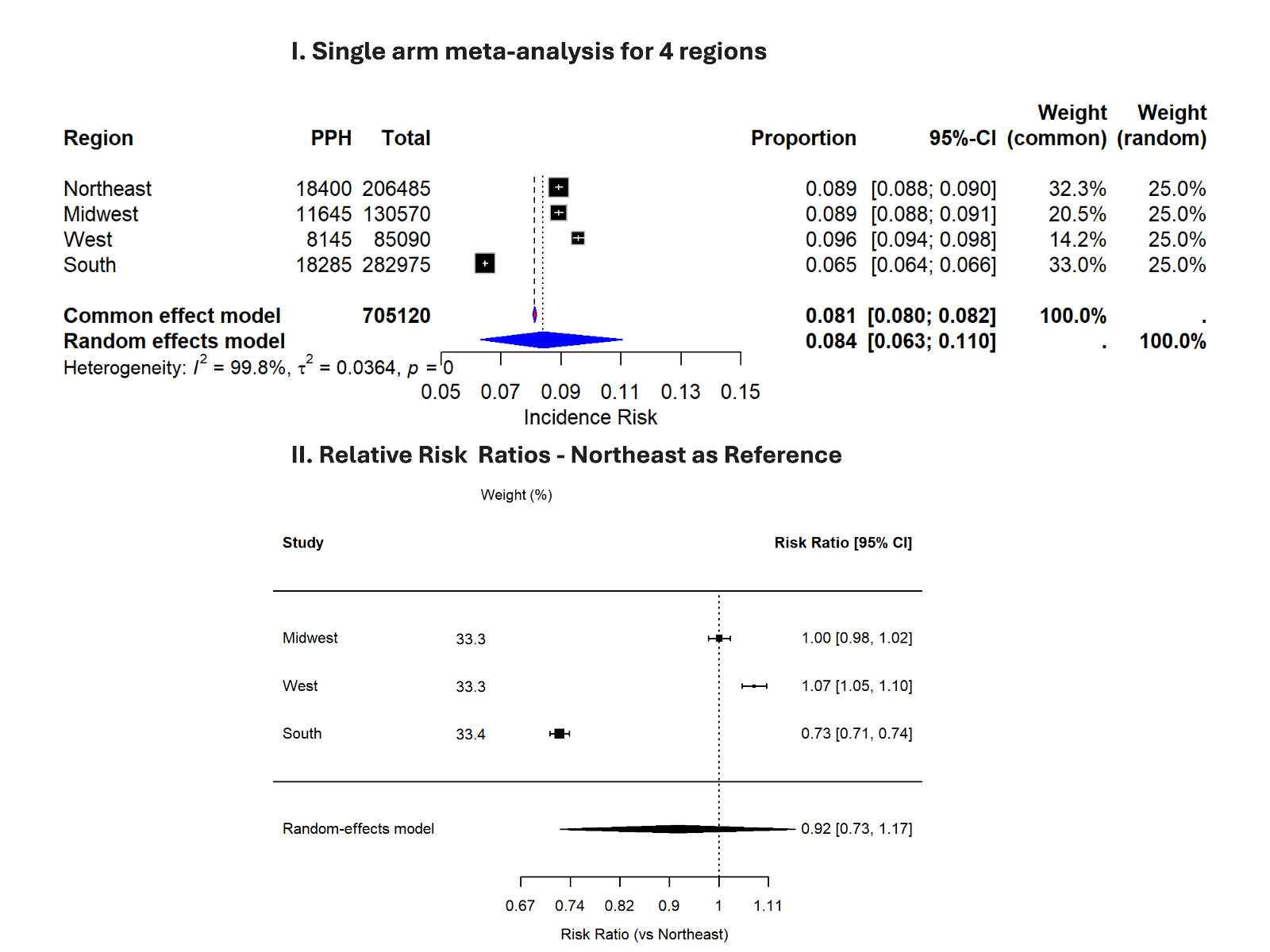

Supplement: Supplementary file 1 — Supplementary Information [file 44401_2026_88_MOESM1_ESM.docx]
